# Forensic Dental Age Estimation: Development of New Algorithm Based on the Minimal Necessary Databases

**DOI:** 10.3390/jpm12081280

**Published:** 2022-08-04

**Authors:** Monika Bjelopavlovic, Ann-Katrin Zeigner, Jochen Hardt, Katja Petrowski

**Affiliations:** 1 Department of Prosthetic Dentistry, University Medical Center of the Johannes Gutenberg-University Mainz, Augustusplatz 2, 55131 Mainz, Germany; 2 Private Practice S12 Fachzahnärzte Mehlingen, Schulstraße 12, 67678 Mehlingen, Germany; 3 Department of Medical Psychology and Medical Sociology, University Medical Center of the Johannes Gutenberg-University Mainz, Duesbergweg 6, 55131 Mainz, Germany

**Keywords:** dental age estimation, Demirjian staging, forensic expert, layperson, wisdom teeth

## Abstract

Objectives Dental age determination relies on the presence of wisdom teeth, which can be assigned to specific age ranges according to their stage of development. The purpose of this study is to highlight the applicability of the Demirjian staging of layman compared to expert, as well as the inclusion of all four wisdom teeth in the overall assessment, in order to emphasize and critically highlight a precise age estimation in clinical practice, especially in the case of agenesis or the presence of less than all four wisdom teeth. **Material and Methods:** In this study, dental age determination is performed and compared by a trained layperson and an expert using 385 orthopantomograms. The radiographs of known chronological age from male patients in the age range of 11–22 years were acquired from the University Medical Center Mainz. All four wisdom teeth were radiologically viewed if present. Demirjian staging with stages A–H was applied, and regression analysis was performed. **Results:** The relationship between mineralization of wisdom teeth (18, 28, 38 and 48) and age was linear for all teeth (*p* < 0.01), except for tooth 18 (*p* = 0.02). Comparing the prediction of the four teeth individually revealed that the lower teeth gave better predictions than the upper ones (R^2^ ≥ 0.50 vs. R^2^ < 0.50). **Conclusions:** For clinical use, the mandibular wisdom teeth should be preferred when performing dental age estimation using the Demirjian staging method. As a result of the present analysis, two ways of determining dental age by wisdom teeth can be suggested. One is to take only tooth 38, with the formula: age = 3.3 + 0.73 × mineralization of tooth_38. The second recommendation would be to take tooth_48. If both are unavailable, the formula would be age = −0.5 + 0.94 × mineralization tooth_28. Utilizing tooth 18 would not lead to more precise results.

## 1. Introduction

Dental age determination is used worldwide in cases of missing documents or unknown body findings and is intended to be a non-invasive and precise methodology for determining the most probable chronological age. Interdisciplinary cooperation between forensic medicine, radiology and dentistry is standard for a more accurate approach according to the “Arbeitsgemeinschaft für forensische Altersdiagnostik” (AGFAD) guidelines. The determination of chronological age based on human biological structures is used for a wide range of issues. Hereby, forensic age determination is an important approach both in the discovery of unknown dead bodies to establish identity and in the age assessment of asylum seekers without paper or identity documents [1,2,3]. It is described in the literature that a tendency to overestimate age in relation to the chronological age can be observed and that gender-related and population-specific reference tables are decisive for an accurate age determination [4,5,6,7,8,9]. Age regulations playing an important role within criminal proceedings. The legally relevant age limits of 14, 18 and 21 have an impact on the overall sentence. For each age determination, three specialist disciplines (forensic medicine, radiology and dentistry) join to form an overall expert opinion in accordance with the AGFAD standard. This working group for forensic age estimation established guidelines for age estimation inside and outside the criminal proceedings [10,11,12]. The dental age diagnosis includes the examination of the wisdom teeth according to Demirjian’s staging method and divides the development of the teeth into eight stages: A–H [11,13,14]. These stages refer to the development of the crown and root of every tooth and is analyzed radiologically. The transfer of the stages into age data with the corresponding standard deviation is carried out. This method for the staging-age assignment is gender- and population-specific based on reference studies with corresponding reference tables [14,15,16]. This reference ensures a differentiated view of tooth development and associated stages according to population- and nutrition-specific criteria [17,18,19]. The mean ages assigned to the stages are given with the respective standard deviation and indicate a minimum and maximum age in each case. The indication of the highest minimum age from the interdisciplinary findings is relevant to the overall opinion [20]. The expert’s dental age estimation should always refer to the person’s stated age and, thereby, consider the probability of the indicated age as well as the standard deviations of the radiological age estimation [13,21,22]. According to the established expert standard, an overestimation of age should be avoided at all costs and the legal principle “in dubio pro reo” (when in doubt rule for the accused) should also be applied in dental age diagnostics. Consequently, dental age estimation based on the wisdom teeth is described as a reliable and consistent methodology in the literature, compared to determining age based on bone development. Nevertheless, different stages of tooth development could be detected in the same biological organism [23]. The delayed development of wisdom teeth frequently observed in the maxilla is discussed as well as a different speed of development in the left and right quadrant (1. + 4. vs. 2. + 3. quadrant) [24]. The wisdom tooth is the only tooth that develops post-pubertally and can provide information about dental age within the limits of legal interest and is therefore used widely for age determination. However, the literature shows completed growth (stage H) with a great variety in different populations [25,26]. Overall, the literature suggests that the structural development of wisdom teeth exhibits a constancy that is more independent of environmental factors and nutritional habits, compared to other bone developments of the human skeleton, but the variance of different populations must be considered and appropriate reference tables used [13,27,28]. The age estimation by original Demirjian’s technique has evolved in the last decades and has been applied to different populations, and in this study, it leads to an overestimation— expert by 1.4 years on average and lay by 1.9 years. Additionally, precision is only moderate, the R^2^s of the technique are 0.49 for experts and 0.48 for lays (R^2^ = R squared is the explained variance of the target variable and lies between 0 and 1). Even though the Demirjian’s staging method is implemented frequently in the literature/forensic practice, it is still unclear how many wisdom teeth and which ones the evaluator needs in order to produce reliable results. The aim of the present paper is to examine the relation between chronological age and mineralization of the wisdom teeth on a per tooth basis and considering all available teeth.

Our study was based on the following hypotheses:

1. The dental age determination of the layman differs from that of the expert.
2. The wisdom teeth of the mandible lead to a more precise dental age determination related to chronological age.
3. By developing a regression function, a more accurate dental age determination is possible.

## 2. Materials and Methods

### 2.1. Subject Group

From the existing database of the University Medical Center Mainz, 385 orthopantomograms were randomly selected by one dentist (not the expert and not the lay). All radiographs were taken in the context of clinical therapy and with known chronological age of the patients who were undergoing dental, orthodontic and/or dental surgical therapy at the time of imaging. Only radiographs of male patients between the ages of 11 and 22 (date of birth: 1 January 1997–1 January 2006) were selected, which was clearly recorded at the time of radiography. The selection of only male patients was a result of our clinical experience, as we almost exclusively performed dental age estimation in male asylum seekers due to orders of youth departments or prosecutor’s offices. The legally relevant limits in criminal proceedings were thus to be depicted, and in accordance with the AGFAD guidelines, a dental age diagnosis based on the wisdom tooth stages according to Demirjian was to be performed. In this context, the German federal criminal police (BKA) published statistics in 2021 that pointed out that 86.4% of all suspects in the population of asylum seekers were male [29]. All of the included radiographs were available in digital form in the SIDEXIS^®^ XG 2.63 program (2016 Sirona Dental Systems GmbH, Wals, Austria). The forensic expert and the trained layperson viewed the 385 radiographs within three months (1 January–31 March 2020) and assessed the stages of the wisdom teeth according to Demirjian (stages A–H). This procedure took place independently and without consultation. The computer and the viewing monitor were the same and within the possibilities of the program; a change in contrast, magnification and brightness was equally possible and usable for both. We followed the ethical standards of the local ethics committee, and general consent of every participant was obtained.

Out of the 385 subjects, a total of 209 had all four wisdom teeth examined, while 167 had at least one wisdom tooth missing or unable to measure mineralization. A total of 1267 wisdom teeth were analyzed.

### 2.2. Inclusion Criteria

- Orthopantomograms taken between 1 January–31 March 2020;
- Male patients;
- Minimum one wisdom tooth present.

### 2.3. Exclusion Criteria

- No syndromal previous diseases, tumor;
- Female.

### 2.4. Design

The forensic expert had 9 years of professional dental experience. The activity in forensic age determination corresponded to 6 professional years. The trained layperson had a degree in dentistry of 9 semesters and was trained based on literature from the AGFAD guidelines and five example radiographs provided in self-study of 1 semester. Both observers were female and evaluated the radiographs in the same room on the same computer and viewing monitor. They were allowed to regulate contrast in the program SIDEXIS^®^ XG 2.63 program (2016 Sirona Dental Systems GmbH, Wals, Austria) individually. The radiographs were viewed within three months (1 January–31 March 2020) on different days but within the same time frames (1 p.m.–4 p.m.). The environment followed the guidelines of Dagassan and Lübbers [30]. All radiographs were viewed with focus on the wisdom teeth. Demirjian’s staging method was performed, while dividing every wisdom tooth into stages A–H. Every stage is linked to an age with standard deviation according to the AGFAD group standards. The data were collected in an excel list that contained only the patient code. Then, the information about the chronological_age was complemented. At the end of the evaluation by expert and lay, we had an excel list with patient code, dental age and the chronological age.

### 2.5. Equipment

- Computer operating on Windows 10 (2015, Redmond, Washington, DC, USA);
- Viewing monitor (calibration according to DIN 6868-157);
- SIDEXIS^®^ XG 2.63 program (2016 Sirona Dental Systems GmbH, Wals, Austria).

### 2.6. Statistical Analysis

First, four bivariate OLS regressions were performed predicting the chronological age by the mineralization degree of the respective tooth (18, 28, 38, 48). Tests of nonlinearity were performed by adding a squared term. To examine the additional value of measuring more than one tooth, multiple imputations using regressions with predictive mean matching (pmm, k = 3) were performed in 100 data sets because about 20% of the individual teeth were either missing or mineralization could not be determined. Results were combined by Rubins rules or by simply averaging the statistics over the 100 imputed data sets for parameters where Rubins rules were not available. Significance level α was set to 0.01 for all tests; accordingly, 99% confidence intervals were calculated. Analyses were performed in STATA V16.

Derivation with calculation example:

Statistics reported in Table 1, Table 2 and Table 3 and in the figures were based on regression analyses using the ordinary least square (OLS) method developed by Galton [31]. Here, the age (Y was estimated by a weighted result of Demirjians Classification (X) plus a constant. For tooth 48, it was: Y = 0.72 × X + 3.41 (Table 2, last line). If for instance a person would have an estimated age after Demirjian of 18.2, we would have corrected this to be 0.72 × 18.2 + 3.41 = 16.5. In Table 3, a somewhat more elaborated method was used. Since more than one tooth became involved in the formulas, some persons had missing data on some teeth. They were substituted by multiple imputation by predictive mean matching. Here, the missing data were substituted in 100 new data sets using the mineralisation of the present tooth by an algorithm introducing random error. Again, simple OLS regressions were performed separately in each of the 100 data sets, results were combined via Rubins Rules [32].

## 3. Results

**Figure 1:**
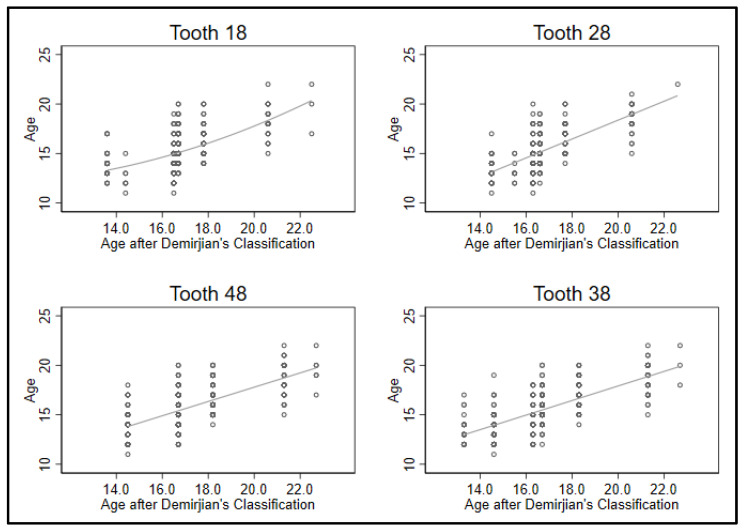
Observed and estimated values for age and tooth mineralization.

**Figure 2:**
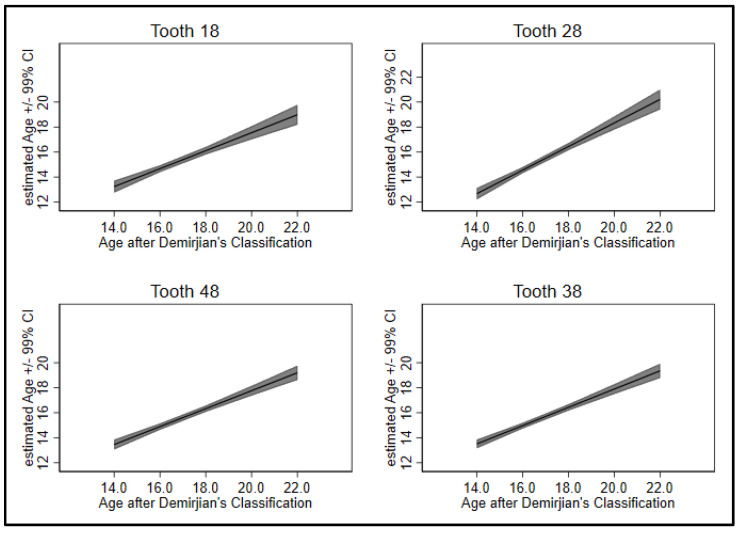
Estimated values for age and tooth mineralization with confidence intervals.

In this study, the dental age estimation using the Demirjian’s staging method leads to an overestimation from the expert by 1.4 years on average and from lay by 1.9 years. Looking at the four possible wisdom teeth 18, 28, 38 and 48, results for the individual teeth predicting age returned that the relation between mineralization and age were linear (*p* < 0.01) for all teeth **except for tooth 18** (*p* = 0.02). Tooth 18 showed a minimal increase in the association with higher mineralization (Figure 1, upper left; Figure 2). Hence, only linear predictions were utilized in further analyses.

Comparing the prediction of the four teeth individually revealed that the **lower teeth gave better predictions than the upper ones** (R^2^ ≥ 0.50 vs. R^2^ < 0.50, Table 2, last column). Particularly, tooth 18 performed poor (R^2^ = 0.35). Comparing the regression coefficients showed similar values for teeth 18, 38 and 48 (beta ~ 0.72), tooth 28 has a steeper gradient of beta = 0.94. Standard errors are 0.06 for the upper teeth and 0.04 for the lower teeth. Constants are close to 3.3 for teeth 18, 38 and 48, -0.5 for tooth 28 (columns 1–3, Table 2).

Examination of the the prediction of the four teeth combined was conducted in the imputed data sets by utilizing all four teeth yields in an R^2^ of 0.57, with coefficients displayed in Table 3, first line. The coefficient of tooth 18 is even slightly negative, and teeth 28 and 48 showed the highest coefficients. Excluding tooth 18 leads to the model displayed in line 2 of Table 3, still yielding a R^2^ = 0.57, and not containing a negative coefficient. Additionally, excluding tooth 38 leads to the model displayed in line 3 of Table 3, still yielding R^2^ = 0.56. Combining teeth 28 and 38 yields R^2^ of 0.55, and combining teeth 38 and 48 to R^2^ of 0.53 (Table 3, line 4 and 5), which is initially astonishing because one could expect that combining the best performing wisdom tooth (38) with another one would lead to a better result.

## 4. Discussion

As a result of the present analysis, two ways of determining age by wisdom teeth can be suggested. One is to take only one tooth; then, we would suggest calculating the dental age simply as age = 3.3 + 0.73 × Demirjian mineralization of tooth_38. If available, tooth_48 would also be possible. If both are unavailable, the formula would be dental age = −0.5 + 0.94 × Demirjian mineralization tooth_28. Utilizing tooth 18 would lead to considerably less precise results. The second way would be to use two teeth, and then, we would suggest calculating dental age = 0.51 × Demirjian mineralization tooth_28 × 0.41 × Demirjian mineralization tooth_48. Coefficients for other combinations of teeth are displayed in Table 3. The gain in precision utilizing more than two teeth is minimal.

On first sight, it may be surprising that the tooth which provided the best estimate alone (38) did not perform optimally when included in the formula to estimate age by two teeth. The answer is that tooth 38 has correlations to 28 and 48 of r = 0.81 and r = 0.93; hence, combining it with one of the two does not add as much information as combining teeth 28 and 48.

Moness Ali et al. performed regression analysis using Demirjian’s staging method in Egyptian children between 3 and 10 years. They could prove that precising the Demirjian staging method in a population could lead to a more accurate dental age estimation, which leads to a better approach to chronological age [33]. In the clinical application of dental age determination, the legal age limits are relevant for social benefits, penalties and obligation to care for minors vs. adults. Therefore, we studied the Demirjian’s staging method and regression analysis on a population within these age groups.

1. The dental age determination of the layman differs from that of the expert.
  This hypothesis could be confirmed, although the difference was only slight: Demirjian’s staging method leads to an overestimation in our study—expert by 1.4 years on average, lay by 1.9 years.
2. The wisdom teeth of the mandible lead to a more precise dental age determination related to chronological age.
  This hypothesis could be confirmed. Comparing the four teeth showed a more precise prediction of chronological age while looking at the lower teeth (R^2^ ≥ 0.50 vs. R^2^ < 0.50, Table 2, last column).
3. By developing a regression function, a more accurate dental age determination is possible.
  This hypothesis could be confirmed. Using regression function gives a more precise approach to chronological age while performing Demirjian’s staging method on wisdom teeth.

The present study has the following limitations: (1) The age range in the present sample is restricted to 11–22. Since predictions are completely linear, it would be possible to apply the suggested formula to younger or older individuals, but only in a limited way, say 10–24. To providing a wider age range, additional data would be needed. (2) Data rely solely on male subjects. Previous research showed small difference between females and males (e.g., Willems). Formulas presented here should not be applied to estimate female’s age. (3) Orthopantomograms (OPTG) were included that showed at least one wisdom tooth, not all four in all available radiographs. This reflects the clinical situation in age diagnostics but limits the information output in this study.

Overall, estimates for the lower teeth are similar, consistent, and can therefore be assumed as reliable. Estimates for the upper teeth are not. Tooth 18 explained considerably less variance than all other teeth, and tooth 28 had a different gradient than the other three. It is likely that the upper teeth are more difficult to measure than the lower ones, because more bone is distracting in the X-ray. Analyzing additional data would help to obtain more precise estimates for the upper teeth as well.

Given these limitations, the suggested formulas provide an easy way to estimate age from the wisdom teeth 28, 38, and 48, tooth 18 still needs further examination. The precision is comparable or even better than Demirjian’s technique when at least one lower tooth can be involved. Furthermore, X-ray of one or two teeth is sufficient, resulting in reduced radiation for the subject examined [34]. However, this statement should be substantiated with studies of the applicability of the staging method to dental films, as it is based on dental panoramic radiographs. Limiting the examination to only one region would require the knowledge of the absence or presence of the wisdom teeth, although they are frequently not visible during clinical examination [35,36]. Therefore, positioning the tube for ideal acquisition does not appear to be clinically applicable. Consequently, the gold standard of dental panoramic radiographs for dental age estimation seems to be undisputed. The formula elaborated here should be applied to an independent and larger collective to test the applicability and to allow the inclusion of one wisdom tooth or certain combinations for a more precise result.

A population-specific age classification of the stages should be used in any case, and interdisciplinary collaboration with forensic medicine and radiology should be emphasized as a key factor for an accurate overall conclusion.

## 5. Conclusions

Within the limitations of this study, the following conclusions are drawn:

1. The biological age is linear to the mineralization stages of the wisdom teeth except for tooth 18 in this study.
2. As a result of this study, we would recommend a preferred use of the lower wisdom teeth for dental age estimation.
3. The formulas that emerged in our analysis show a more precise approach to the chronological age. One might speculate that future research should test this result on a larger population.

## Figures and Tables

**Table 1:** Sample description.

Variable	Valid Obs.	Missing Obs.	Mean	SD
Age chronological	385	0	15.45	2.17
Age estim. by expert *	385	0	16.85	1.87
Age estim. by lay *	385	0	17.33	1.99
Mineralization Tooth				
18	307	78	16.79	1.78
28	316	69	16.75	1.50
38	333	52	16.71	2.16
48	311	74	17.05	2.14

* Age estimation with the Demirjian’s technique.

**Table 2:** Linear prediction of age when utilizing mineralization of each tooth individually.

Variable	Beta	SE_beta_	Cons	R^2^
Tooth 18	0.72	0.06	3.17	0.35
Tooth 28	0.94	0.06	−0.52	0.47
Tooth 38	0.73	0.04	3.27	0.52
Tooth 48	0.72	0.04	3.41	0.50

Note: Calculations were performed on samples between *n* = 307–333, see Table 1.

**Table 3:** Linear prediction of age when utilizing mineralization of several teeth combined.

Variables	Beta	SE_beta_	Cons.	R^2^
Teeth 18, 28, 38, 48	−0.10, 0.54, 0.16, 0.31	0.12, 0.14, 0.12, 0.10	0.16	0.57
Teeth 28, 38, 48	0.46, 0.14, 0.31	0.09, 0.12, 0.10	0.13	0.57
Teeth 28, 48	0.51, 0.41	0.08, 0.06	−0.04	0.56
Teeth 28, 38	0.49, 0.41	0.09, 0.07	0.35	0.55
Teeth 38, 48	0.38, 0.37	0.11, 0.10	2.85	0.53

Note: Calculations were performed on 100 imputed samples with *n* = 385, see text.

## Data Availability

The data from this study were part of the dissertation paper from A.-K.Z. Data can be seen in Table 1, Table 2 and Table 3 and Figure 1 and Figure 2.
